# Supplementary material for: Content-rich biological network constructed by mining PubMed abstracts
Source: BMC Bioinformatics. 2004 Oct 8;5:147. doi: 10.1186/1471-2105-5-147 (PMC528731; doi:10.1186/1471-2105-5-147)
Supplement: Additional File 2 — The original results of the above study (non-essential files are deleted to keep the file size under the limit set by BMC bioinformatics). [file 1471-2105-5-147-S2.bz2 › chilibotAdditionalFile2/dip10/293ID7033218E1125/html/NADB_NADA.html]

 


 **NADB** and **NADA** 
  
Found 14 abstracts in PubMed, retrieved 10.  
 

 What does Google say? 
 PDF only 
| .edu only 

---

**Interactive relationship** (e.g. stimulation, inhibition, etc)

**Neutral relationship**- It has been reported that this gene and  **nadA**  are regulated by a positive regulatory protein encoded in the  **nadB**  region.  Ref: 3305482 J Bacteriol, 1987
- In several bacteria, the gene in the position occupied by TM1643 encodes an aspartate oxidase  **NadB** , which synthesizes iminoaspartate as a substrate for  **NadA** , the next enzyme in the pathway.  Ref: 12496312 J Biol Chem, 2003
- Quinolinic acid is synthesized in E. coli by the enzymes L aspartate oxidase  [ **NADB** ]  and quinolinate synthase A, the genes of which are named  **nadB**  and  **nadA** .  Ref: 2187483 Biol Chem Hoppe Seyler, 1990

**Non-interactive relationship** (e.g. studied together, co-existance, homology, etc.)

- As NAD levels increase within the cell, the affinity of NadR for the operator regions of  **nadA** ,  **nadB** , and pncB increases, repressing the transcription of these target genes.  Ref: 2198247 J Bacteriol, 1990
- In Salmonella typhimurium, de novo synthesis of NAD is regulated through the transcriptional control of the  **nadA**  and  **nadB**  loci.  Ref: 2198247 J Bacteriol, 1990
- Transfer of cultures from aerobic to anaerobic conditions resulted in the partial derepression of both  **nadA**  and  **nadB** .  Ref: 3934331 J Gen Microbiol, 1985
- Cloning and characterization of quinolinate synthesis genes  **nadA**  and  **nadB** .  Ref: 2841129 Eur J Biochem, 1988
- The  **nadA**  17 unit map position and  **nadB**  55 units genetic loci involved with quinolinic acid biosynthesis were both found to be regulated by the product of a nadR locus 99 units in a repression derepression manner while nadC 3 units expression appeared constitutive at the transcriptional level.  Ref: 3934331 J Gen Microbiol, 1985
- Finally, our data definitely exclude the possibility that other enzymes are involved in the biosynthesis of quinolinic acid in E. coli, since it is possible to synthesize quinolinic acid from l aspartate, dihydroxyacetone phosphate, and O 2 by using only  **nadA**  and  **nadB**  gene overexpressed products.  Ref: 10648170 Protein Expr Purif, 2000
- Mutations at the nadI locus affect expression of the first two genes of NAD synthesis,  **nadA**  and  **nadB** , which are unlinked.  Ref: 3275606 J Bacteriol, 1988
- The nadI protein regulates transcription of the  **nadA**  and  **nadB**  biosynthetic genes and modulates activity of the NMN permease.  Ref: 1991724 J Bacteriol, 1991
- Common null mutations nadI show constitutive high level expression of the  **nadB**  and  **nadA**  genes.  Ref: 3275606 J Bacteriol, 1988
- The presence of cAMP cAMP receptor protein was essential for the complete derepression of  **nadA**  while no effect was evident upon  **nadB** .  Ref: 3934331 J Gen Microbiol, 1985
- The nadI locus encodes a repressor protein acting on the unlinked  **nadA**  and  **nadB**  genes.  Ref: 3275606 J Bacteriol, 1988
- High copy number vectors, carrying either gene  **nadA** ,  **nadB**  or  **nadA**   **nadB** , increased quinolinate production 1.5 fold, 2.0 fold and 15 fold respectively.  Ref: 2841129 Eur J Biochem, 1988
- Increases in nadAB transcription directly correlated with decreases in intracellular NAD P levels, and kinetic studies indicated that the NAD analogue 6 amino NAD was ineffective in repressing either  **nadA**  or  **nadB** .  Ref: 3934331 J Gen Microbiol, 1985
- The two genes,  **nadA**  and  **nadB** , responsible for quinolinate biosynthesis from aspartate and dihydroxyacetone phosphate in Escherichia coli were cloned and characterized.  Ref: 2841129 Eur J Biochem, 1988
- Rare nadIs mutations cause constitutive low level expression of  **nadB**  and  **nadA** .  Ref: 3275606 J Bacteriol, 1988
